# Fibroblast Growth Factor Receptors-1 and -3 Play Distinct Roles in the Regulation of Bladder Cancer Growth and Metastasis: Implications for Therapeutic Targeting

**DOI:** 10.1371/journal.pone.0057284

**Published:** 2013-02-26

**Authors:** Tiewei Cheng, Beat Roth, Woonyoung Choi, Peter C. Black, Colin Dinney, David J. McConkey

**Affiliations:** 1 Department of Urology, The University of Texas M.D. Anderson Cancer Center, Houston, Texas, United States of America; 2 Department of Cancer Biology, The University of Texas M.D. Anderson Cancer Center, Houston, Texas, United States of America; 3 Experimental Therapeutics Academic Program, The University of Texas-Graduate School of Biomedical Sciences (GSBS) at Houston, Houston, Texas, United States of America; 4 Department of Urologic Science, The University of British Columbia, Vancouver, British Columbia, Canada; China Medical University, Taiwan

## Abstract

Fibroblast growth factor receptors (FGFRs) are activated by mutation and overexpressed in bladder cancers (BCs), and FGFR inhibitors are currently being evaluated in clinical trials in BC patients. However, BC cells display marked heterogeneity in their responses to FGFR inhibitors, and the biological mechanisms underlying this heterogeneity are not well defined. Here we used a novel inhibitor of FGFRs 1–3 and RNAi to determine the effects of inhibiting FGFR1 or FGFR3 in a panel of human BC cell lines. We observed that FGFR1 was expressed in BC cells that also expressed the “mesenchymal” markers ZEB1 and vimentin, whereas FGFR3 expression was restricted to the E-cadherin- and p63-positive “epithelial” subset. Sensitivity to the growth-inhibitory effects of BGJ-398 was also restricted to the “epithelial” BC cells and it correlated directly with FGFR3 mRNA levels but not with the presence of activating FGFR3 mutations. In contrast, BGJ-398 did not strongly inhibit proliferation but did block invasion in the “mesenchymal” BC cells in vitro. Similarly, BGJ-398 did not inhibit primary tumor growth but blocked the production of circulating tumor cells (CTCs) and the formation of lymph node and distant metastases in mice bearing orthotopically implanted “mesenchymal” UM-UC3 cells. Together, our data demonstrate that FGFR1 and FGFR3 have largely non-overlapping roles in regulating invasion/metastasis and proliferation in distinct “mesenchymal” and “epithelial” subsets of human BC cells. The results suggest that the tumor EMT phenotype will be an important determinant of the biological effects of FGFR inhibitors in patients.

## Introduction

Bladder cancer (BC) is the fifth most common cancer in Western countries. Bladder cancers can be divided into two major subgroups that possess distinct pathological, clinical, and molecular characteristics [1], [2]. Most BCs (70%–80%) are low grade, non-muscle invasive papillary (“superficial”) tumors (NMIBCs) that rarely progress, so patients with this form of cancer have a very good prognosis. On the other hand, patients with muscle-invasive bladder cancers (MIBCs) have a much poorer prognosis (<50% 5-year survival) [1], [2]. MIBCs often progress to become metastatic, and patients with metastatic disease have a dismal 5-year survival rate of less than 5%. Consequently, identifying the molecular mechanisms involved in BC invasion and metastasis and identifying therapeutic strategies that target these processes are very high priorities in ongoing research.

Fibroblast growth factor receptors (FGFRs) are very attractive candidate targets in both subsets of BCs [3]. At least two thirds of NMIBCs contain activating FGFR3 mutations that result in ligand-independent receptor dimerization and constitutive downstream signal transduction [4], [5], [6], [7], and in vitro studies have established that FGFR inhibitors block proliferation in normal urothelial cells that overexpress these receptors [8], [9]. Although the frequency of activating FGFR3 mutations in MIBCs is much lower (<25%), many of them express high levels of FGFR3 and other FGFRs [3], [10], [11]. In addition to promoting proliferation, FGFRs have been implicated in the regulation of epithelial-to-mesenchymal transition (EMT), invasion, and anchorage-independent growth in BC cells [11].

BGJ-398 is a selective inhibitor of FGFRs 1, 2, and 3 that was synthesized using a novel chemical approach [12]. It exhibits IC_50_’s of approximately 5 nM against wild-type FGFRs and the most common mutant form of FGFR3 that is expressed in BCs (S249C) [12]. An initial characterization of the compound’s growth inhibitory effects in a panel of 8 human BC cell lines revealed marked heterogeneity in responses, where it displayed IC_50_’s of 5–30 nM in half of the cell lines and IC_50_’s of over 1 µM in the other half [12]. The observed heterogeneity is consistent with results obtained using a distinct chemical inhibitor [13], but the molecular basis for this heterogeneity remains unclear. We therefore initiated the present study to obtain a better understanding of the effects of FGFR inhibition in BC cells, with the goal of identifying biological mechanisms and biomarkers that could be used to prospectively identify FGFR-dependent tumors. Our results reveal distinct, EMT-related roles for FGFR3 and FGFR1 in driving proliferation and invasion that have important implications for the development of FGFR inhibitor-based therapies in patients.

## Materials and Methods

### Chemicals and Reagents

BGJ-398 was generously provided by Novartis. For in vitro studies, BGJ-398 was reconstituted in DMSO at a stock concentration of 10 mmol/L and stored at −20°C. The BGJ-398 stock was diluted in medium just prior to use so that the concentration of DMSO never exceeded 0.1%. For in vivo studies, BGJ-398 was dissolved in 10% Tween-80.

### Tumor Cell Lines and Culture Conditions

Cell lines were obtained from the University of Texas MD Anderson Cancer Center Bladder SPORE Tissue Bank, and their identities were confirmed by DNA fingerprinting using the AmpFlSTR® Identifiler® Amplification (Applied Biosystems) or AmpFlSTR® Profiler® PCR Amplification (Applied Biosystems) protocols. All cell lines were maintained as monolayers in modified Eagle’s MEM supplemented with 10% fetal bovine serum, vitamins, sodium pyruvate, L-glutamine, penicillin, streptomycin, and nonessential amino acids at 37°C in a 5% CO_2_ incubator.

### Animals

Female athymic nude mice (NCr-nu) were purchased from the National Cancer Institute. The mice were housed under specific pathogen-free conditions in the Animal Core Facility at The University of Texas M. D. Anderson Cancer Center. The facility has received approval from the American Association for Accreditation of Laboratory Animal Care and in agreement with current regulations and standards of the U.S. Department of Health and Human Services, the U.S. Department of Agriculture, and the NIH. The mice used in these experiments were 6 to 8 weeks old.

### FGFR3 Mutation Analyses

DNA was isolated from BC cell lines using a genomic DNA extraction kit (Qiagen). PCR was performed to amplify exons 7 and 10 using AmpliTaq Gold DNA polymerase (Applied Biosystems) and the primers 5′-CGGCAGTGGCGGTGGTG- GTG-3′(sense) and 5′-AGCACCGCCGTCTGGTT GGC-3′ (antisense) for exon 7 (23) and 5′-CCTCAACGCCCATGTCTTT-3′ (sense) and 5′-AGGCAGCTCAGAACCTGGTA-3′ (antisense) for exon 10 (purchased from Sigma Genosys). The following cycling variables were used: 95°C for 10 min, then 35 cycles of 95°C for 30 s, 65°C (exon 7) or 58°C (exon 10) for 30 s, and 72°C for 30 s, followed by a final incubation at 72°C for 10 min (23). Unincorporated primers and deoxynucleotides were removed using shrimp alkaline phosphatase and exonuclease I (U.S. Biochemical). Products were analyzed by Big Dye Terminator Cycle Sequencing (Applied Biosystems), and the data were analyzed with Sequencing Analysis 3.0 software (Applied Biosystems).

### RNAi-mediated Knockdown of FGFR3, FGFR1 or bFGF

UM-UC14 and RT4 were transfected with small interfering RNAs (siRNAs) targeting FGFR3 and FGFR1 using Oligofectamine (Invitrogen) according to the manufacturer’s instructions. Targeted oligonucleotides (sequence) and a non-targeting control were purchased from Ambion. Total RNA was collected 48 hours after transfection and analyzed by RT-PCR to confirm target knockdown. In parallel, siRNA transfected cells were harvested at 48 hours and analyzed using the cell proliferation and cell cycle assays described below. UM-UC3 and UM-UC13 were transduced with lentiviral short hairpin RNAs (shRNAs)(Open Biosystems). Cells were continuously cultured for 5∼7 days in 10% MEM containing puromycin. Total RNA was collected after selection and analyzed by RT-PCR to confirm target knockdown. Stable knockdown cells were maintained in puromycin and used in the MTT and Boyden chamber invasion assays described below.

### Immunoblotting Analyses

Cells were harvested at ∼75% to 85% confluence and lysed. Protein concentrations were measured using the Bradford assay (Bio-Rad Laboratories, Hercules, CA). Lysates were boiled in sample buffer (62.5 mmol/L Tris-HCl (pH 6.8), 10% (w/v) glycerol, 100 mmol/L DTT, 2.3% SDS, 0.002% bromophenol blue) for 5 minutes and cooled on ice for 5 minutes. Samples were separated on 8% or 12% SDS-PAGE gels at 110 V in electrophoresis buffer (25 mmol/L Tris-HCl (pH 8.3), 192 mmol/L glycine, 0.1% SDS) and then electrophoretically transferred onto methanol-prewetted polyvinylidene difluoride (PVDF) membranes in transfer buffer (25 mmol/L Tris-HCl, 192 mmol/L glycine, 20% methanol) for 1 hour at 100 mV. The membranes were incubated in blocking buffer (TBS: 10 mmol/L Tris-HCl (pH 8.0), 150 mmol/L NaCl, 5% nonfat milk) for 1 hour at room temperature while shaking and then rinsed once briefly with TBS-T (TBS containing 0.1% Tween-20). The membranes were incubated with primary antibodies diluted 1∶1000 in blocking buffer overnight, washed, and then incubated with second antibodies (anti-mouse or anti-rabbit immunoglobulin, horseradish peroxidase–linked F(ab)2 fragment from mouse) diluted 1∶8,000 in blocking buffer for 1 hour at room temperature while shaking. Immunoreactive proteins were detected using enhanced chemiluminescence (Amersham Biosciences, Piscataway, NJ) according to the manufacturer’s instructions.

### Gene Expression Profiling

Gene expression profiling was performed on a panel of 30 human BC cell lines using the Illumina platform. For each cell line, mRNA was generated from a single log-phase culture. RNA purity and integrity were measured using a NanoDrop ND-1000 and an Agilent Bioanalyzer, and only high quality RNA was used for cRNA amplification. Biotin-labeled cRNA was prepared using the Illumina RNA amplification kit (Ambion, Inc, Austin, TX), and amplified cRNA was hybridized to Illumina HT12 V4 chips (Illumina, Inc., San Diego, CA). After they were washed, the slides were scanned with IScan (Illumina, Inc.). Signal intensities were quantified with GenomeStudio (Illumina, Inc.), and quantile normalization was used to normalize the data. BRB ArrayTools version 4.2 developed by National Cancer Institute [14] was used to analyze the data. To observe the expression patterns of differentially expressed genes, specific gene expression values, adjusted to a mean of zero, were used for clustering with Cluster and TreeView [15].

### MTT Assays

Cells (5×10^3^) were plated in 96-well plates and allowed to adhere for 24 hours before they were incubated with or without increasing concentrations of BGJ-398 for 48 h or 5 days. MTT (3-(4,5-dimethylthiazol-2-yl)-2,5-diphenyltetrazolium bromide) assays were used to measure relative cell numbers based on conversion of MTT to formazan in viable cells. Fifty µl MTT dissolved in PBS (50 µg/ml) was added to each well and plates were incubated for 3 hours. The medium was then removed and 100 µl DMSO was added to each well to lyse cells and solubilize the formazan. A standard micro-plate reader was used to determine the absorbance (600 nm). Each experimental data point represents average values obtained from six replicates and each experiment was performed at least twice.

### Real-time Reverse Transcriptase PCR Analyses

Cells were harvested at 75% to 85% confluence and total RNA was isolated using mirVANA™ miRNA Isolation Kit (Ambion, Life Science, CA). FGFRs and other genes of interests were analyzed by Taqman-based real-time PCR (ABI PRISM 7500; Applied Biosystems). The comparative CT method was used to determine relative gene expression for each target gene; the cyclophilin A gene was used as internal control to normalized the amount of amplifiable RNA. Taqman primers was purchased from the manufacture (Applied Biosystem, CA) as follows: E-cadherin; Hs00170423_m1, TP63; Hs00978343_m1, ZEB1; Hs00232783_m1, Vimentin; Hs00185584_m1, FGFR1; Hs00915142_m1, FGFR2; Hs01552926_m1, FGFR3; Hs00179829_m1, FGFR4; Hs01106908_m1, bFGF; Hs00266645_m.

### Cell Cycle Analyses

Cells were plated in 6-well plates and maintained in 10% FBS MEM for 24 hours. Cells were then exposed to various concentrations of BGJ-398 for 48 hours or grown another 24 hours (reaching ∼75% to 85% confluence) to analyze the effects of FGFR3 or FGFR1 knockdown. Cells were harvested by trypsinization and pelleted by centrifugation. The pellets were then resuspended in PBS containing 50 µg/mL propidium iodide, 0.1% Triton X-100, and 0.1% sodium citrate. Propidium iodide fluorescence was measured by fluorescence-activated cell sorting (FL-3 channel, Becton Dickinson, Mountain View, CA) using the instrument’s cycle analysis software.

### Boyden Chamber Invasion Assays

Invasion chambers containing Matrigel-coated polyethylene terephthalate membranes with 8 µm pores were purchased from BD Science in a 24-well plate format. Cells (2.5×10^5^) were released from tissue culture flasks using EDTA (1 mmol/L), centrifuged, suspended in a serum free medium and placed in the upper compartments of invasion chambers. Thirty percent fetal bovine serum medium was placed in the lower compartments as a chemoattractant and invasion assays were carried out for 48 h. Each cell line was plated in triplicate. To examine cell invasion after exposure to BGJ-398, cells that had not invaded were removed and the cells on the lower surface of the filter were stained with Diff-Quick (American Scientific Products, McGaw Park, IL). Invasive activity was measured by counting the cells that had migrated to the lower side of the filter. To evaluate invasion after silencing FGFR1 or bFGF, membranes were removed after incubation for 48 hours at 37°C and stained in propidium iodide (Sigma-Aldrich) without removing cells from the upper surfaces of the membranes. The filters were mounted on glass slides and analyzed by confocal microscopy at 100× magnification. The planes of focus were adjusted so that the cells that had not invaded could be distinguished from the invaded cells and counted in 8 independent fields. Invasive activity was measured by calculating ratios of invaded to noninvaded cells.

### Archorage Independent Growth Assay

UM-UC3 and UM-UC13 wild type or bFGF/FGFR1 silenced cells were plated at 1×10^4^ cells per well in 6-well-plates supplemented with 10% FBS MEM containing 0.6% agar. Cells were allowed to grow for 2 weeks. Images were acquired using an Olympus IX inverted-phase contrast microscope. The total numbers of colonies per random view (100×) and the average diameter of colonies per random view (100×) were determined using a SliderBook image analyzer.

### Orthotopic Xenograft Experiments

The human BC cell line UM-UC-3 was transduced with a lentiviral vector encoding luciferase (luc) and red fluorescent protein (RFP; mCherry) as described previously [16]. After stable transduction with the luc-RFP reporter, cells were sorted by Fluorescence Activated Cell Sorting (FACS) using an Influx High-Speed sorter (BD Biosciences). Luciferase activity was quantified in vitro using D-luciferin (150 µg/mL) and the IVIS bioluminescence system (Xenogen Co.). To produce tumors in nude mice, subconfluent cultures of labeled UM-UC3 were lifted with trypsin, mixed with 10% FBS MEM, centrifuged at 1,200 rpm for 5 min, washed in PBS, and resuspended in HBSS. Cells were then injected orthotopically into the bladder wall at a concentration of 5×10^5^/50 µL using a lower laparotomy. Mice bearing metastases were euthanized 5 to 8 weeks after tumor cell injection, the lymph node and distant metastases were excised, cut into small pieces using scalpels, exposed to 1% trypsin for 20 minutes, centrifuged (1,200 rpm for 5 min), and cultured in 10% supplemented MEM. After FACS sorting, the recycled cells were subconfluently cultured and reinjected at a concentration of 2×10^5^/50 µL HBSS as described above. Thus, tumor cell recycling was performed three times in order to select a highly metastatic UM-UC3 subpopulation which develops metastases in ∼75% of mice. For our therapy experiment, we injected the 4^th^ cycle of recycled UM-UC3 at a concentration of 2×10^5^/50 µL. Mice with detectable tumor growth at the time of the first imaging (5 days after injection) were randomized into two groups (n = 7/group).

### In vivo Bioluminescence Imaging

Bioluminescence imaging was conducted on an IVIS 100 imaging system with Living Image software (Xenogen) as described elsewhere [16]. In brief, animals were anesthetized before imaging with a 2.5% isoflurane/air mixture and injected s.c. with 15 mg/mL of luciferin potassium salt in PBS at a dose of 150 mg/kg body weight. A digital gray-scale animal image was acquired and a pseudocolored image was overlaid representing the spatial distribution of detected photons emerging from active luciferase. Signal intensity was quantified as the sum of all detected photons within the region of interest per second, separately counting each primary tumor and each metastatic site.

### Collection of Primary Tumors and Circulating Tumor Cells (CTCs)

Forty days after injection, when animals in the control group became moribund, mice were anesthetized with isoflurane as described above. To measure the number of CTCs, the maximal amount of blood (600–1200 µl) was collected by cardiac puncture using 1 ml syringe, 22 gauge needle, and heparin-coated collection tubes as described previously [17]. Mice were then euthanized with carbon monoxide. Tumors were excised, and samples were either formalin fixed and embedded in paraffin, embedded in OCT (Miles, Inc), or frozen rapidly in liquid nitrogen and stored at −80°C for RNA and protein extraction. For further blood processing, red blood cells were lysed twice for 5 min with 1 ml ACK lysis buffer (Invitrogen), and centrifuged for 5 min at 1200 rpm in Eppendorf tubes. The pellet was finally lysed and further processed for total RNA isolation using the mirVANA™ miRNA Isolation kit (Ambion, Life Science). For Real-time PCR analysis, PCR technology (Step One; Applied Biosystems) was used together with TaqMan® Gene Expression Assays (Applied Biosystems). Absolute quantification was used to generate cycle threshold (CT) values for human specific HLA-C primer (Hs00740298_g1) for each sample. RT-PCR analysis of the blood samples (in triplicates) was run together with standard isolates (0, 2, 20, 200, 2000, and 20,000 UM-UC3 cells in 100 µl mouse blood). CT values of the standards were used to create a standard curve for UM-UC3 CTC, and the number of CTCs of each blood sample was calculated accordingly.

## Results

### Relationship between E-cadherin and bFGF/FGFR Expression in UC Cells

**Figure 1:**
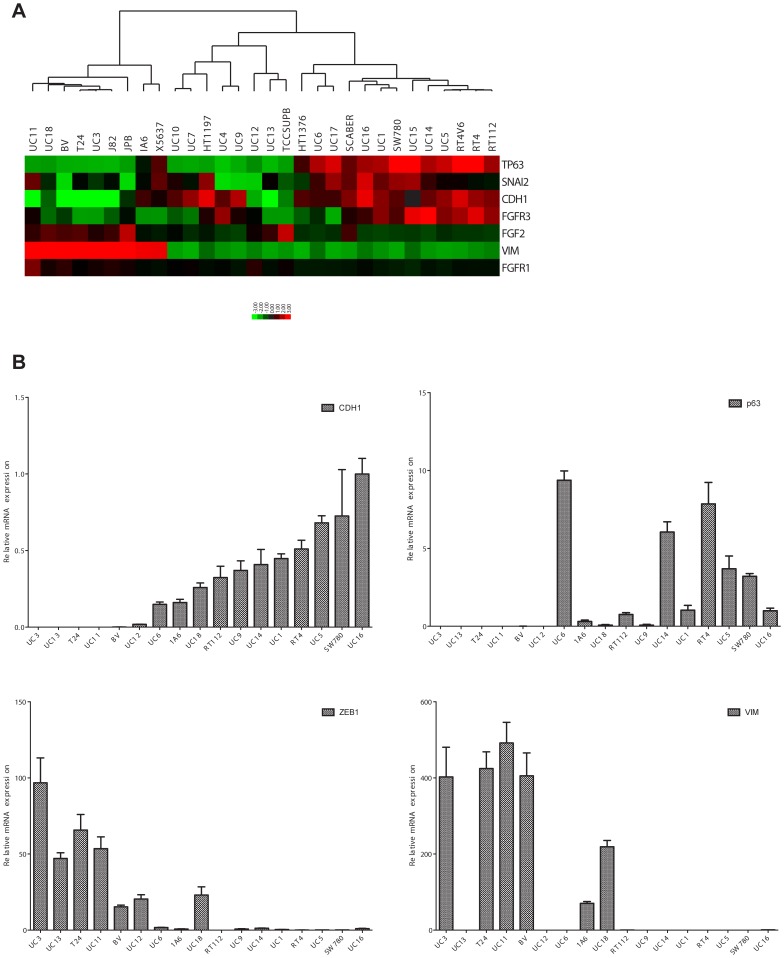
Expression of FGFR1, FGFR3, and bFGF in distinct subsets of human BC cell lines. A. Correlation of FGFR1 and FGFR3 with canonical EMT markers. mRNA levels were measured using whole genome mRNA expression profiling (Illumina platform). The heat map depicts the expression of FGFR1, FGFR3, FGF2 (bFGF), p63 (TP63), E-cadherin (CDH1), Slug (SNAI2), and vimentin. B. Quantitative analysis of EMT marker expression. Relative levels of the “epithelial” markers E-cadherin (CDH1) and p63, and the “mesenchymal’ markers ZEB1 and vimentin were measured by quantitative real-time RT-PCR.

We analyzed the expression of the 4 FGFRs and the dominant cancer-associated ligand (FGF-2/basic FGF) at the mRNA level in a panel of 30 UC cell lines by whole genome expression profiling (Illumina platform). Expression of FGFR3 correlated directly with expression of p63 [18], [19] and E-cadherin [20](Fig. 1A), indicating that FGFR3 is expressed by the “epithelial” subset of BC cells. Conversely, the expression of FGFR1 correlated more directly with the “mesenchymal” marker vimentin (Fig. 1A). We used quantitative real-time reverse transcriptase PCR (RT-PCR) to more accurately define the patterns of expression of “epithelial” and “mesenchymal” markers across the panel of cell lines. The results are displayed in Figure 1B, where we organized the BC cell lines in all of the panels according to their relative expression of the canonical “epithelial” marker E-cadherin (Fig. 1B, upper left panel) [20]. The expression of E-cadherin correlated directly with expression of p63 and inversely with expression of ZEB1 and vimentin, demonstrating that the “epithelial” and “mesenchymal” markers are expressed in a largely non-overlapping fashion in the BC lines (Fig. 1B). Only two of the cell lines (1A6 and UM-UC18) co-expressed “epithelial” and “mesenchymal” markers (Fig. 1B).

**Figure 2:**
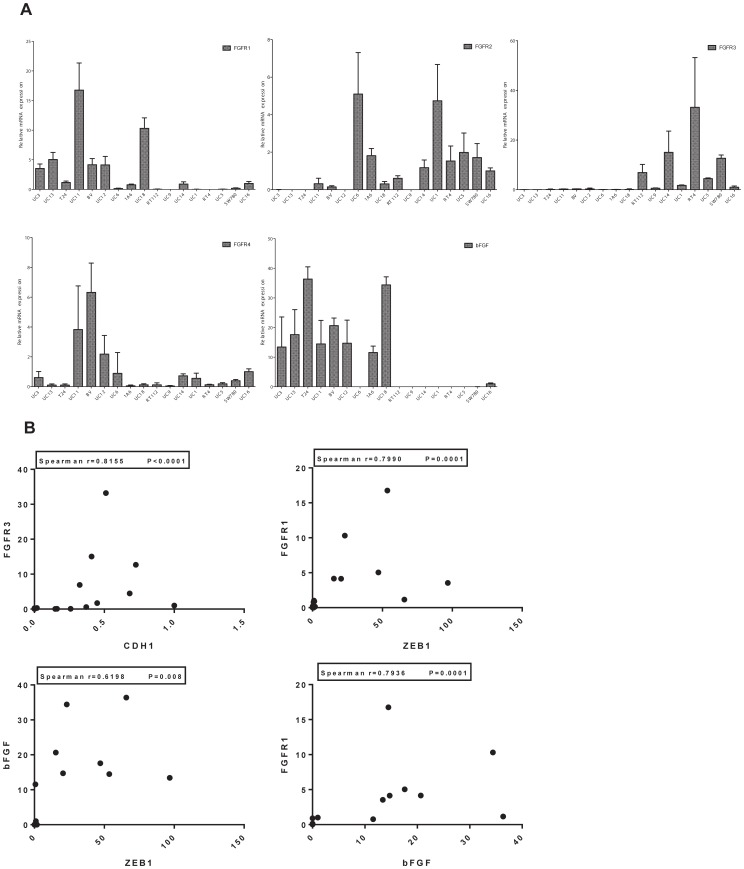
Relationship between FGFR/bFGF expression and EMT. A. Expression of FGFRs 1–4 and bFGF in relationship to E-cadherin expression. The relative mRNA levels were measured by quantitative real-time RT-PCR. The cell lines in each panel are organized by relative E-cadherin expression (low to high, from left to right; see Fig. 1B). B. Scatterplots depicting the relationships between FGFR1, bFGF, FGFR3, and EMT marker expression. Nonparametric correlation analyses were used to evaluate the relationships between FGFR3 and E-cadherin (CDH1) expression, FGFR1 and ZEB1 expression, bFGF and ZEB1 expression, and bFGF and FGFR1 expression. Correlation coefficients and p values are indicated on the figure.

We then measured expression of FGFRs 1–4 and FGF-2 by quantitative RT-PCR (Fig. 2A). Confirming the gene expression profiling results, FGFR1 was expressed primarily by cells within the “mesenchymal” subset (UM-UC3, UM-UC13, T24, BV and UC12)(Fig. 2A top left), whereas FGFR3 expression was concentrated within the “epithelial” cells, with RT4 having the highest expression followed by UM-UC14, SW780 and RT112 (Fig. 2A, top right). FGFR2 also appeared to be somewhat enriched in the “epithelial” and FGFR4 in the “mesenchymal” cells, respectively, but their levels of expression were much lower than the levels of FGFR3 or FGFR1 (mean of 36 cycles versus 30 cycles of PCR), consistent with previous findings [10]. Finally, the “mesenchymal” cells expressed higher levels of FGF2 (bFGF) than did the “epithelial” cells (Fig. 2A). We confirmed that FGFR3 expression was enriched in the “epithelial” and FGFR1 and bFGF expression was enriched in the “mesenchymal” cell lines at the protein level by immunoblotting (Figure S1). Using nonparametric correlation analyses, we confirmed that FGFR3 expression correlated strongly and directly with expression of E-cadherin (Spearman r = 0.8155, p<0.0001, Fig. 2B) and inversely with expression of the “mesenchymal” markers (Fig. S2). Conversely, expression of FGFR1 and bFGF strongly and directly correlated with expression of ZEB1 (Spearman r = 0.799, p = 0.0001 for FGFR1 and r = 0.6198, p = 0.008 for bFGF) (Fig. 2B). In addition, bFGF and FGFR1 expression correlated directly with each other as expected (Fig. 2B). We then examined whether the observed differences in mRNA expression translated into differential expression at the protein level in a subset of the cell lines by immunoblotting. We found that FGFR3 but not FGFR1 was expressed in the epithelial UM-UC14, RT4 and RT112 cells, whereas FGFR1 but not FGFR3 was expressed in mesenchymal UM-UC3, UM-UC12 and UM-UC13. FGF-2 was expressed in all 6 cell lines, but the mesenchymal cells expressed more FGF-2 than the epithelial” cells did. Together, these data support the idea that FGFR3 and bFGF/FGFR1 probably function in non-overlapping epithelial and mesenchymal subsets of BC cells.

### Effects of BGJ-398 on Cell Proliferation

**Figure 3:**
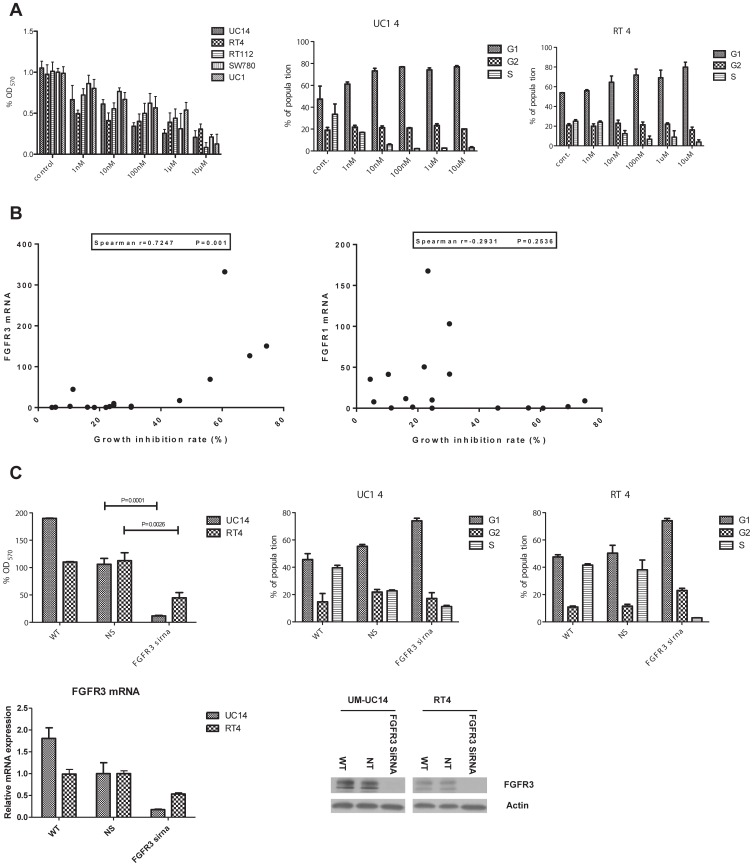
FGFR3 levels predict sensitivity to BGJ-398-induced cell cycle arrest. A. Effects of BGJ-398 on cell proliferation in the drug-sensitive cells. In the left panel, cells were incubated for 48 h in the presence of the indicated concentrations of BGJ-398 and cell growth was measured by MTT reduction. Mean ± SEM, n = 6. In the center and right panels, UM-UC14 or RT4 cells were incubated with the indicated concentrations of BGJ-398 and the percentages of cells within each cell cycle quadrant were quantified by propidium iodide staining and FACS analysis. Mean ± SEM, n = 3. B. Sensitivity to the anti-proliferative effects of BGJ-398 correlates with FGFR3 expression but not with the presence of activating FGFR3 mutations. The level of growth inhibition observed after 48 h exposure to 1 µM BGJ-398 (as measured in MTT assays) was correlated with the relative level of FGFR3 (left panel) or FGFR1 (right panel) mRNA expression in a panel of 17 human BC cell lines.. C. Effects of FGFR3 knockdown on cell proliferation. Left panel: UM-UC14 or RT4 cells were transiently transfected with either non-targeting (NT) or FGFR3-specific siRNAs and cell growth was measured at 48 h by MTT reduction. Mean ± SEM, n = 6. Center and right panels: UM-UC14 or RT4 cells were transiently transfected with either non-targeting (NT) or FGFR3-specific siRNAs and percentages of cells within each phase of the cell cycle were quantified by propidium iodide staining and FACS analysis. Mean ± SEM, n = 3. Lower panel: the efficiency of FGFR3 silencing was measured by quantitative RT-PCR and immunoblotting.

Previous studies concluded that FGFR inhibitors block proliferation in some human BC cells in vitro [12], [13]. We therefore tested the effects of BGJ-398 on proliferation in 17 BC cell lines to characterize the extent of the heterogeneity in drug sensitivity. We incubated the cells with increasing concentrations of BGJ-398 for 48 hours and measured cytotoxicity and growth arrest using MTT assays. We identified 5 cell lines (UM-UC14, SW780, RT4, RT112 and UM-UC1) that were drug-sensitive as defined by ≥50% growth inhibition at drug concentrations of 1 µmol/L or lower (Fig. 3A left panel and data not shown). To determine the relative contributions of growth arrest and cell death to these effects, we exposed the UM-UC14 and RT4 cells to increasing concentrations of BGJ-398 for 48 hours and directly measured cell cycle arrest and apoptosis by propidium iodide staining and FACS analysis. In both cell lines increasing concentrations of BGJ-398 produced increases in the percentages of cells in the G1 phase and parallel decreases in the percentages of cells in S phase. Specifically, the percentages of cells within the G1 phase increased from 47.5% and 54% to 74.2% and 69.1%, while percentages of cells in S phase decreased from 33.5% and 25% to 2.7% and 8.8% in the BGJ-398-exposed UM-UC14 and RT4 cells, respectively (Fig. 3A). On the other hand, BGJ-398 did not induce apoptosis in either cell line at concentrations below 10 µM (data not shown). Therefore, BGJ-398 exerts primarily cytostatic effects on BC cells in vitro.

We then examined the relationship between BGJ-398 sensitivity and the presence of activating FGFR3 mutations. Using exon sequencing we identified 5 cell lines within our panel that contained activating FGFR3 mutations (UM-UC6, UM-UC14, UM-UC15, UM-UC16 and UM-UC17) (Fig. S3). Strikingly, only one of the FGFR3-mutant cell lines (UM-UC14) was also BGJ-398 sensitive. On the other hand, we observed a good correlation between FGFR3 mRNA expression and drug sensitivity (Spearman r = 0.7247 p = 0.01) (Fig. 3B left panel), whereas there was no correlation between sensitivity to BGJ-398 and FGFR1 expression (Spearman r = −0.2931 p = 0.2536) (Fig. 3B right panel).

Given the non-overlapping patterns of FGFR1 and FGFR3 expression, the results suggested that FGFR3 plays a more important role than FGFR1 in driving proliferation human BC cells. To more directly test this hypothesis, we used RNAi to knock down FGFR1 or FGFR3 in the BGJ-398-sensitive UM-UC14 and RT4 cells and measured the effects on cell proliferation using MTT assays. Quantitative RT-PCR revealed knockdown efficiencies of 50% and over 80% in the RT4 and UM-UC14 cells transfected with FGFR3-specific siRNAs, respectively compared with cells transfected with the non-specific siRNA control, and these results were also confirmed at the protein level by immunoblotting (Fig. 3C lower panel). The corresponding effects on cell proliferation were very similar, in that proliferation was reduced by almost 50% in the RT4 cells and by over 80% in UM-UC14 transfected with the FGFR3 siRNA (Fig. 3C left panel). Cell cycle analyses confirmed that FGFR3 knockdown increased the percentages of cells in the G1 phase and decreased the fractions of cells in S phase (Fig. 3C center/right panels), consistent with the MTT results and the previously observed effects of BGJ-398 [12]. In contrast, knockdown of FGFR1 had no significant effect on proliferation (Fig. S4).

### Effects of BGJ-398 on Invasion

**Figure 4:**
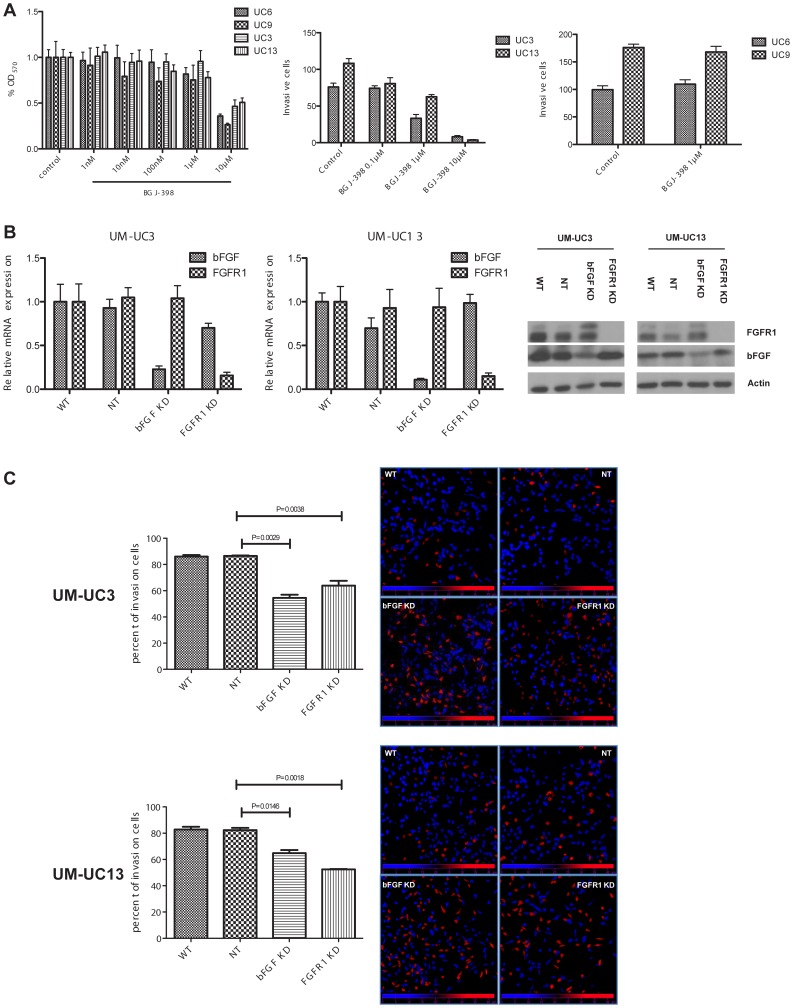
FGFR1 selectively regulates invasion in “mesenchymal” bladder cancer cells. A. Left panel: effects of BGJ-398 on cell growth in two “mesenchymal’ (UM-UC3, UM-UC13) and two “epithelial” (UM-UC6, UM-UC9) cell lines that were found to be resistant to the anti-proliferative effects of the drug. Growth inhibition was measured at 48 h by MTT reduction. Mean ± SEM, n = 6. Center panel: concentration-dependent effects of BGJ-398 on invasion in the UM-UC3 and UM-UC13 cells. Invasion was measured using modified Boyden chambers and standard light microscopy as described in Materials and Methods. Mean ± SEM, n = 3. Right panel: effects of BGJ-398 on invasion in the UM-UC6 and UM-UC9 cells. Note that the drug had no effect on invasion in either cell line. B. Stable knockdown of FGFR1 or bFGF in cells transduced with lentiviral shRNAs. Relative mRNA levels were measured by quantititative real-time RT-PCR and protein levels were measured by immunoblotting. C. Effects of FGFR1 or bFGF knockdown on invasion. Left panels: percentages of cells that invaded through Matrigel in modified Boyden chambers were quantified by propidium iodide staining and confocal microscopy. The right panels display representative confocal images where the nuclei of the cells that invaded are pseudo-colored blue and the cells that did not invade are depicted in red.

Although the “mesenchymal” UM-UC3 and UM-UC13 cells expressed relatively high levels of FGFR1, they were resistant to the anti-proliferative effects of BGJ-398 (Fig. 4A left panel). Because invasion, migration, and metastasis are characteristic features of “mesenchymal” tumor cells [20], we examined the effects of BGJ-398 on invasion in the UM-UC3 and UM-UC13 cells, using two “epithelial”, BGJ-398-resistant cell lines (UM-UC6 and UM-UC9) as controls. We exposed cells to increasing concentrations of BGJ-398 and measured invasion using modified Boyden chambers. BGJ-398 strongly inhibited invasion in the UM-UC3 and UM-UC13 cells in a concentration-dependent manner, whereas it had no effects on invasion in the “epithelial” UM-UC6 and UM-UC9 cells (Fig. 4A center/right panels).

To more directly examine the involvement of FGFR1 and bFGF in the regulation of invasion, we stably silenced their expression in the UM-UC3 and UM-UC13 cells using lentiviral shRNAs, and we used quantitative RT-PCR analyses to confirm that silencing produced over 75% knockdown in all cases, and we confirmed that these effects resulted in reduced protein expression by immunoblotting (Fig. 4B). We then quantified the effects of knockdown on invasion using modified Boyden chambers and confocal microscopy. In the UM-UC3 background the percentage of invading cells was reduced from 85% in the parental cells or cells transduced with a control lentiviral construct to 54.5% in bFGF KD cells (P = 0.0029) and 63.8% in FGFR1 KD cells (P = 0.0038). Similarly, in the UM-UC13 background, the levels of invasion were reduced from 82% in parental cells or cells transduced with the non-targeting lentivirus to 64.8% in the bFGF KD cells (P = 0.0146) and 52.4% in FGFR1 KD cells (P = 0.0018) (Fig. 4C). Together, the data confirm that bFGF and FGFR1 both promote invasion in “mesenchymal” BC cells.

Our observation that FGFR inhibition blocked invasion without affecting proliferation in the cells that express high levels of FGFR1 seemed to contradict previous work implicating FGFR1 in the regulation of cell proliferation [10], [11]. We therefore performed additional experiments to determine whether FGFR1 inhibition produced effects on growth that become more obvious in longer-term assays. Consistent with this idea, knockdown of FGFR1 in UM-UC3 or either bFGF or FGFR1 in UM-UC13 partially inhibited cell growth in 5-day MTT assays (Fig. S5A) and assays that measure colony formation in soft agar (Fig. S5B).

### Effects of BGJ-398 on Tumor Growth and Metastasis

**Figure 5:**
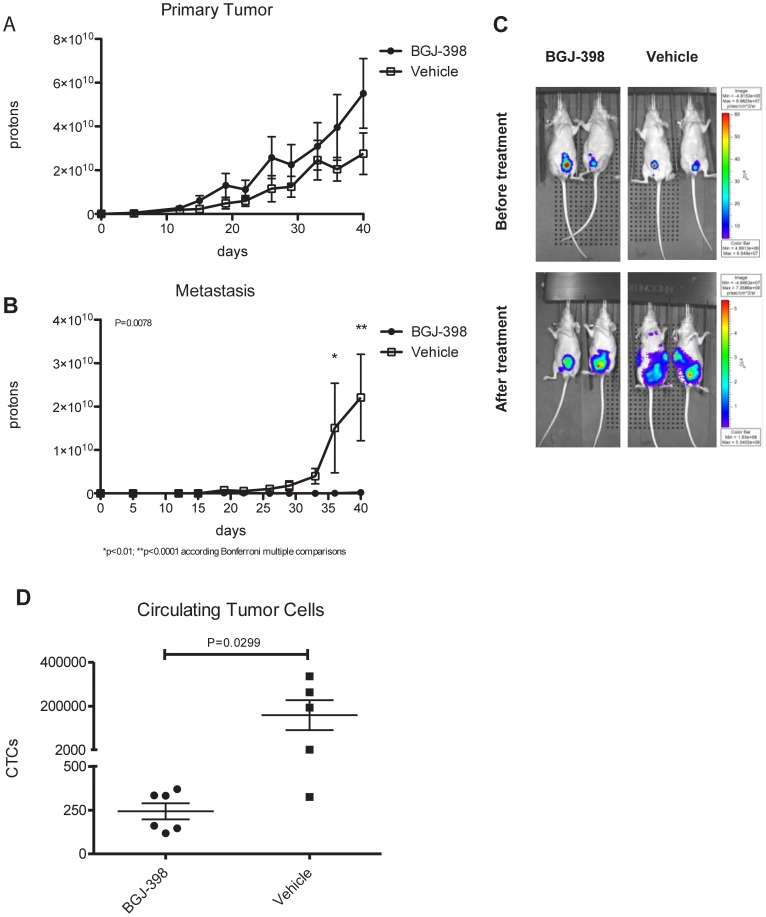
Effects of BGJ-398 on UM-UC3 primary tumor growth and metastasis. A. Effects on primary tumor growth. Luciferase-labelled, orthotopically recycled UM-UC3 cells were implanted into the bladders of nude mice, and tumors were allowed to grow for 8 days prior to initiating therapy with BGJ-398 (daily via oral gavage). Tumor growth was measured biweekly by luciferase imaging. Mean ± SEM from 6 (control) or 7 (treated) mice per group. B. Effects on metastasis. Whole animal metastatic burdens were determined non-invasively by luciferase imaging. Mean ± SEM, n = 6 (control mice) or 7 (treated mice). C. Representative whole body luciferase images taken just prior to the initiation of therapy and at the conclusion of the experiment. D. Effects of BGJ-398 on CTC production. CTC numbers were estimated by measuring human HLA levels in isolated whole blood by quantitative PCR; cell numbers were determined using a UM-UC3 standard curve. The scatterplot displays the results obtained from each animal; the lines denote the mean values for each group.

Although in vitro models are excellent tools for studying molecular mechanisms, the process of cancer metastasis is regulated by tumor-stromal interactions that cannot be modeled well in vitro. Therefore, in order to better define the effects of BGJ-398 on primary tumor growth versus metastasis in “mesenchymal” BC cells, we first isolated a highly metastatic form of UM-UC3 using orthotopic “recycling” in nude mice [21]. We transduced the cells with a lentiviral vector encoding luciferase and red fluorescent protein (RFP), which enabled us to monitor primary tumor growth and metastasis non-invasively by luciferase imaging and to isolate circulating tumor cells (CTCs) by cell sorting. After 3 rounds of recycling, the UM-UC3 cells formed orthotopic tumors in 100% of mice and consistently produced metastases to lymph nodes, lungs, and bone in over 70% of mice. We then implanted 200,000 of the recycled UM-UC3 cells orthotopically in nude mice and initiated therapy with BGJ-398 or vehicle (via oral gavage) once primary tumors were well established (on day 8), monitoring tumor growth and metastasis biweekly by IVIS imaging (Fig. 5). Interestingly, primary tumors in the mice treated with BGJ-398 appeared to grow slightly faster than controls, although the differences in growth rates were not statistically significant (Fig. 5A: P>0.05). In contrast, BGJ-398 strongly inhibited the development of CTCs and metastases. Specifically, 5 out of 7 mice within the control group developed lymph node metastasis by day 15, and two of these subsequently developed bone and lung metastasis at day 36 (Fig. 5C right lower panel). However, we detected only 1 lymph node metastasis in the 7 animals within the BGJ-398 treatment group. When we quantified total metastatic burden using luciferase imaging, the differences between the vehicle and BGJ-398 treatment groups were highly significant (Fig. 5B,C: p = 0.0078). Finally, we quantified the numbers of circulating tumor cells in the mice at the time of sacrifice on day 40 by measuring human HLA-C levels in whole peripheral blood by quantitative PCR. CTC numbers within the control group ranged from 325 to 336,008 cells (mean = 158,977), whereas CTC numbers in the treated group ranged from 160 to 370 (mean = 243.6)(Fig. 5D: p<0.01). Together, the results demonstrate that BGJ-398 had no inhibitory effect on the growth of UM-UC3 primary tumors but did block tumor cell extravasation into the vasculature (as measured by CTC production) and metastasis.

## Discussion

The prevalence of activating FGFR3 mutations [5], [7], [22] and FGFR-1 [10] and -3 [3], [23] overexpression in BCs, coupled with the fact that the mutant forms of FGFR3 drive cell proliferation [9], [24], makes FGFR inhibitors among the most attractive candidates for clinical development [25]. However, it is clear from the results of the preclinical studies that have been published to date that human BC cells display marked heterogeneity in their sensitivities to selective and non-selective FGFR inhibitors [13], [26], [27], [28], which could pose significant challenges to the identification of the appropriate subset(s) of BC patients who will benefit most from FGFR-directed therapy. Based on previous experience in other solid tumors, it seemed likely that the presence of activating FGFR3 mutations identifies FGFR3-dependent BC cells. It also seemed likely that overexpression of either FGFR-1 or FGFR-3 would be linked to FGFR-1 or -3 dependency, respectively. However, our results demonstrate that the presence of an activating FGFR3 mutation does not predict sensitivity to BGJ-398 in established human BC cell lines. Specifically, although some of the BC cell lines that contain activating FGFR3 mutations were highly sensitive to BGJ-398 and other FGFR inhibitors (UM-UC14, 97-7, and MGHU3) [9], [13], [24], [26], [27], [28], several other FGFR3-mutant lines were not (UM-UC6, UM-UC15, UM-UC16, UM-UC17, 94-10, 97-18, J82) [13], [27], and some FGFR3 wild-type cell lines were just as sensitive to BGJ-398 and the other inhibitors as the most sensitive FGFR3 mutant cells (UM-UC1, RT4, RT112, and SW780) [13], [28]. On the other hand, sensitivity to BGJ-398 did correlate closely with FGFR3 mRNA levels (Fig. 3) [13]. Importantly, the cell lines that expressed the highest levels of FGFR1 expressed low levels of FGFR3 and were all relatively resistant to BGJ-398-induced growth arrest when the effects of the drug were measured early (48–72 h), strongly suggesting that FGFR3 is a more important driver of BC cell proliferation than FGFR1. Our data also demonstrate that the effects of BGJ-398 are largely cytostatic (rather than cytotoxic), so clinical “responses” may not be associated with tumor regression, and in future studies we plan to investigate whether FGFR3 inhibition can promote the cytotoxic effects of conventional chemotherapy and/or other investigational agents. Within this context, it is worth noting that all of the available human BC cell lines are derived from muscle-invasive tumors (with the possible exception of RT4), and it seems likely that muscle-invasive BCs that gave rise to the FGFR inhibitor-resistant, FGFR3 mutant cell lines had progressed beyond the point where mutant FGFR3 was essential to maintain proliferation and/or survival. Thus, we suspect that the preclinical data underestimate the potential impact of FGFR3-based therapy in patients with low-grade, non-muscle invasive FGFR3-mutant cancers.

Although our results indicate that FGFR1 plays a less important role than FGFR3 in driving BC tumor cell proliferation, they also strongly suggest that FGFR1 plays crucial roles in invasion and metastasis. Inhibition of FGFR signaling with either BGJ-398 or FGFR1 knockdown resulted in strong suppression of invasion in vitro, and BGJ-398 blocked CTC production and metastasis (without inhibiting primary tumor growth) in mice inoculated with orthotopic, FGFR1-positive UM-UC3 tumors in vivo (Fig. 5). On the surface our conclusions may seem to contradict previous work implicating FGFR1 in tumor cell proliferation in vitro and in primary tumor growth in vivo, especially because some of the experiments employed the same UM-UC3 cell line used here [10]. However, we were able to replicate the effects of FGFR1 knockdown on proliferation in long-term MTT assays and soft agar colony formation, and our conclusion that FGFR1 inhibition does not attenuate primary tumor growth is based on BGJ-398 therapy in established UM-UC3 tumors rather than on experiments with cells stably transduced with an FGFR1 shRNA (where FGFR1 would not be available for tumor establishment), so we are confident that the results of the two studies are consistent with each other. Perhaps a more important question is why inhibition of FGFR3 has stronger effects on proliferation than inhibition of FGFR1, since the effects of both have been clearly linked to inhibition of ERK signaling [10], [11], [27], [28]. We speculate that the differential effects are related to the very distinct epithelial versus mesenchymal biological phenotypes of the FGFR3- versus FGFR1-positive cells and that the “epithelial” cells may be more dependent on autocrine growth factors for proliferation, a conclusion that is consistent with our previous work with inhibitors of the EGF receptor [29], [30]. Clinically, some non-muscle invasive tumors progress to become muscle-invasive and metastatic. If appropriate biomarkers (possibly including FGFR1 and EMT marker expression) can be identified, it is conceivable that these potentially lethal non-muscle invasive tumors could be controlled with FGFR1 inhibitor-based chemoprevention strategies.

Finally, our data show that FGFR3 and FGFR1 are expressed by the “epithelial” and “mesenchymal” subsets of bladder cancer cells, respectively. The strong associations suggested to us that direct cause-effect relationships might exist between them, and we performed some preliminary experiments to address this possibility. We did not detect any changes in FGFR1 and FGFR3 mRNA expression in UM-UC3 cells after knockdown of either ZEB1 or SNAIL (data not shown), strongly suggesting that these canonical EMT transcription factors are not involved in regulating their expression. However, we did observe changes in several EMT regulators in the UM-UC3 or UM-UC13 cells following knockdown of FGFR1 (Fig. S6), consistent with the idea that FGFR1 signaling functions upstream to drive EMT. Parallel studies have implicated PLCγ, ERK, and cyclooxygenase-2 in FGFR1-mediated EMT in BC cells [11]. Defining the transcriptional targets of FGFR1 responsible for mediating EMT will be an important area for future research.

## Supporting Information

Figure S1 *Baseline expression of FGFR1, FGFR3 and bFGF proteins in subsets of epithelial and mesenchymal human bladder cancer cells.* Protein levels in 3 representative “epithelial” (UM-UC14, RT4 and RT112) and 3 “mesenchymal” (UM-UC3, UM-UC12 and UM-UC13) cell lines were measured by immunoblotting.
(TIF)

Figure S2 *Correlation between FGFR/bFGF expression and EMT markers.* The figure displays the results of the correlation analyses. Correlation coefficients are displayed in red, and corresponding p values are depicted in black. Negative correlation coefficients indicate the presence of an inverse relationship between markers.
(TIF)

Figure S3 *FGFR3 mutation status in human bladder cancer cells*. The presence of activating FGFR3 mutations was determined by exome sequencing. Note that among the 5 cell lines within the panel that contain activating mutations, only one (UM-UC14) is sensitive to BGJ-398.
(TIF)

Figure S4 *Effects of FGFR1 knockdown on FGFR1 expression and proliferation in RT4 and UM-UC14 cells*. A. Left panel: UM-UC14 or RT4 cells were transiently transfected with either non-targeting (NT) or FGFR1-specific siRNAs and cell growth was measured at 48 h using MTT. Mean ± SEM, n = 8. Right Panel: the efficiency of FGFR1 silencing by siRNA was determined by quantitative RT-PCR. B. UM-UC14 or RT4 cells were transiently transfected with either non-targeting (NT) or FGFR1-specific siRNAs and percentages of cells within each phase of the cell cycle were quantified by propidium iodide staining and FACS analysis. Mean ± SEM, n = 3.
(TIF)

Figure S5 *Effects of bFGF or FGFR1 knockdown in long-term assays.* A. MTT results obtained in 5-day assays. Mean ± SEM, n = 6. *p<0.05. B. Results obtained in soft agar colony formation assays. The left panels display the numbers of colonies and the right panels colony diameters as determined by measuring colony growth in soft agar. Mean ± SEM, n = 5.
(TIF)

Figure S6 *Effects of bFGF or FGFR1 knockdown on EMT marker expression.* Mean ± SEM, n = 3.
(TIF)
