# Supplementary figures and images for: Fibroblast Growth Factor Receptors-1 and -3 Play Distinct Roles in the Regulation of Bladder Cancer Growth and Metastasis: Implications for Therapeutic Targeting
Source: PLoS One. 2013 Feb 26;8(2):e57284. doi: 10.1371/journal.pone.0057284 (PMC3582560; doi:10.1371/journal.pone.0057284)

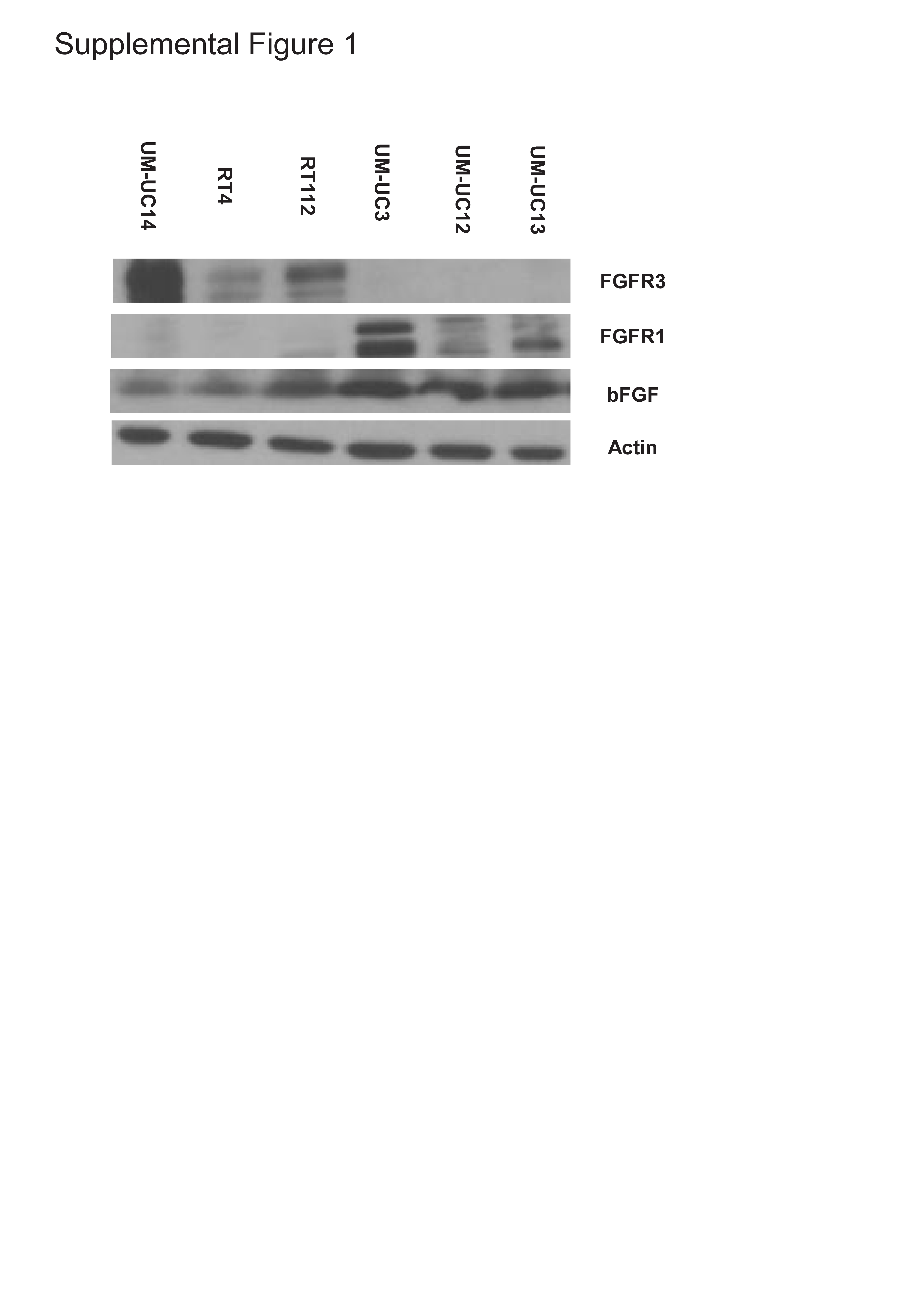

Supplement: Figure S1 — Baseline expression of FGFR1, FGFR3 and bFGF proteins in subsets of epithelial and mesenchymal human bladder cancer cells. Protein levels in 3 representative “epithelial” (UM-UC14, RT4 and RT112) and 3 “mesenchymal” (UM-UC3, UM-UC12 and UM-UC13) cell lines were measured by immunoblotting. (TIF) [file pone.0057284.s001.tif]

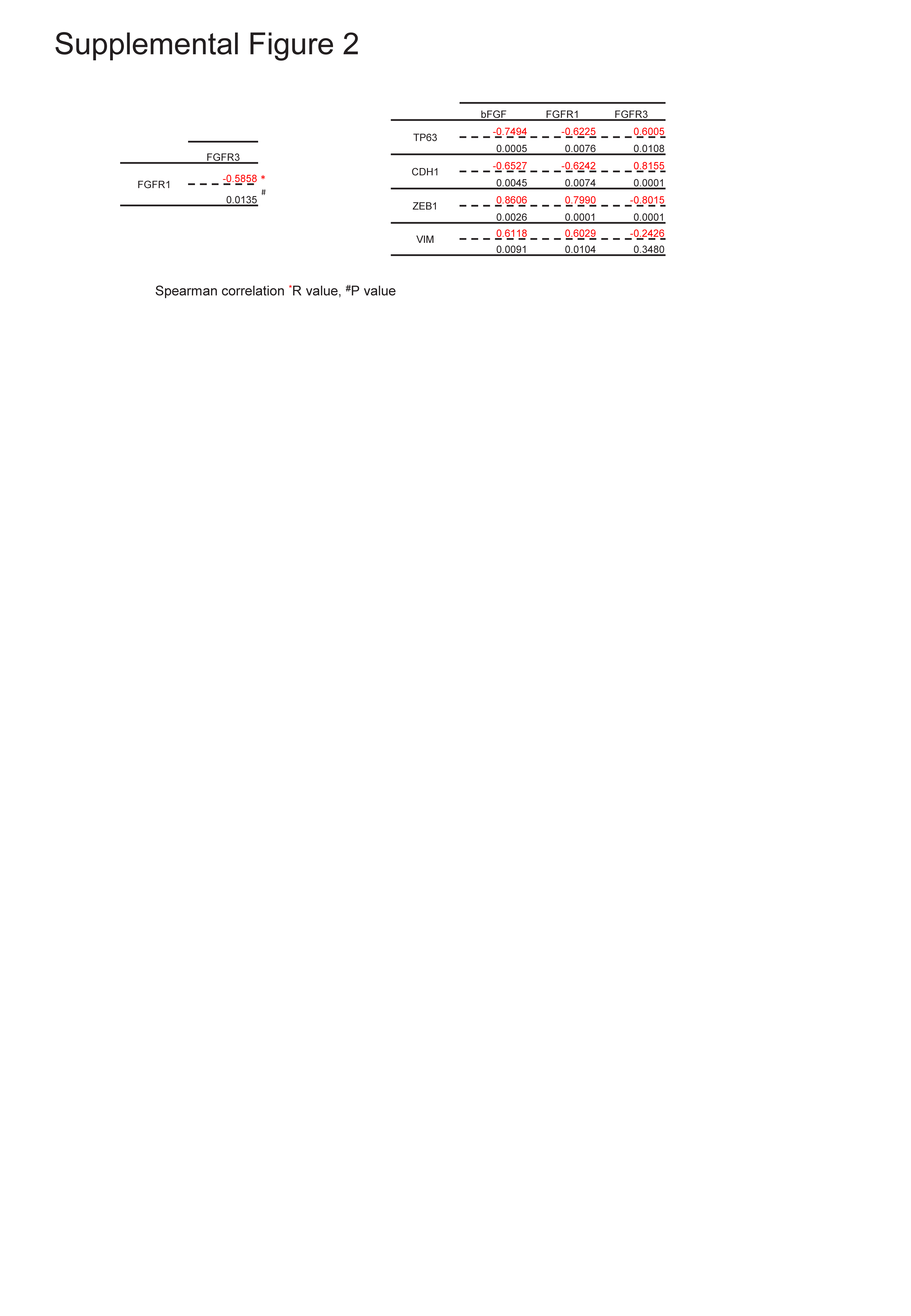

Supplement: Figure S2 — Correlation between FGFR/bFGF expression and EMT markers. The figure displays the results of the correlation analyses. Correlation coefficients are displayed in red, and corresponding p values are depicted in black. Negative correlation coefficients indicate the presence of an inverse relationship between markers. (TIF) [file pone.0057284.s002.tif]

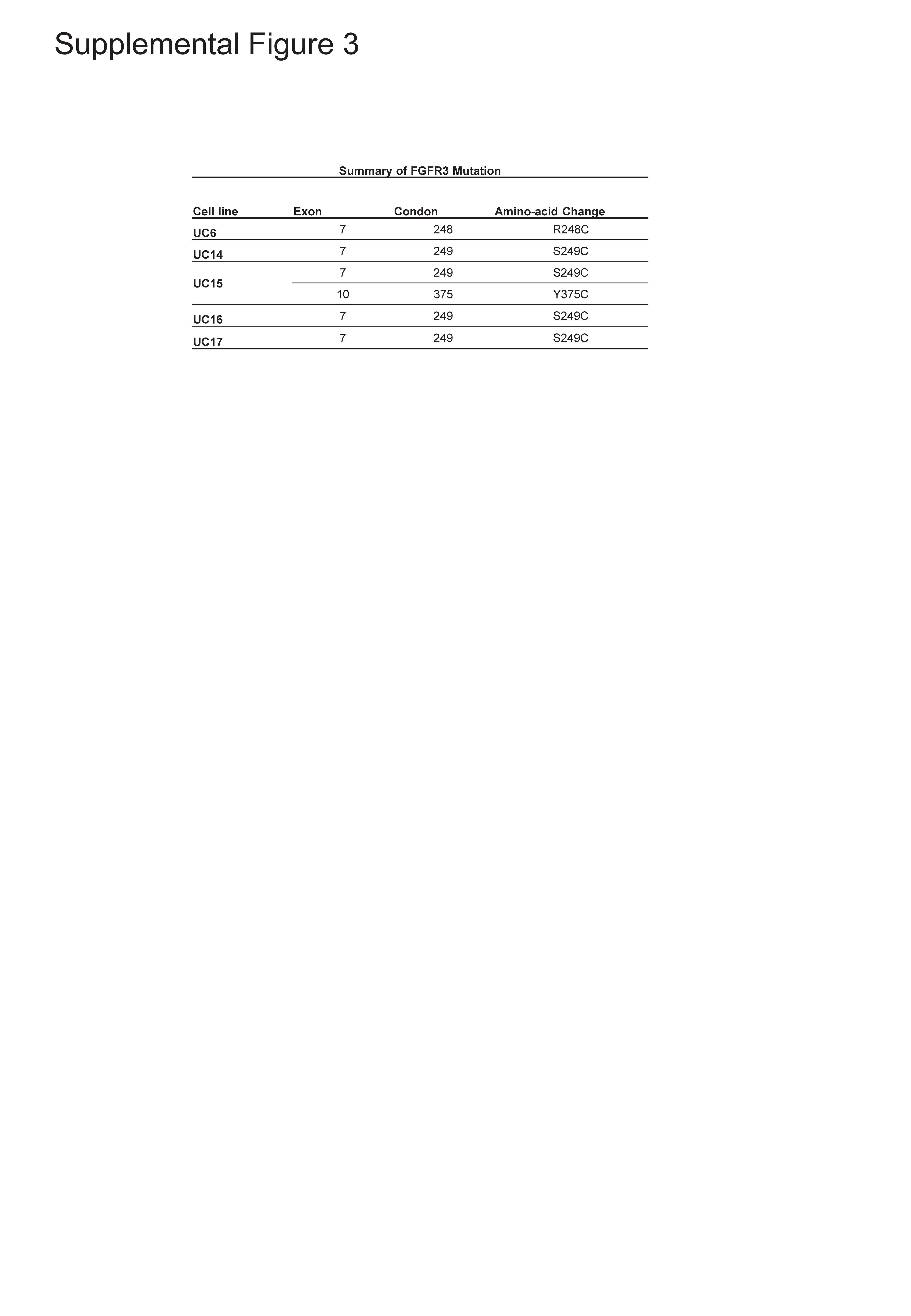

Supplement: Figure S3 — FGFR3 mutation status in human bladder cancer cells. The presence of activating FGFR3 mutations was determined by exome sequencing. Note that among the 5 cell lines within the panel that contain activating mutations, only one (UM-UC14) is sensitive to BGJ-398. (TIF) [file pone.0057284.s003.tif]

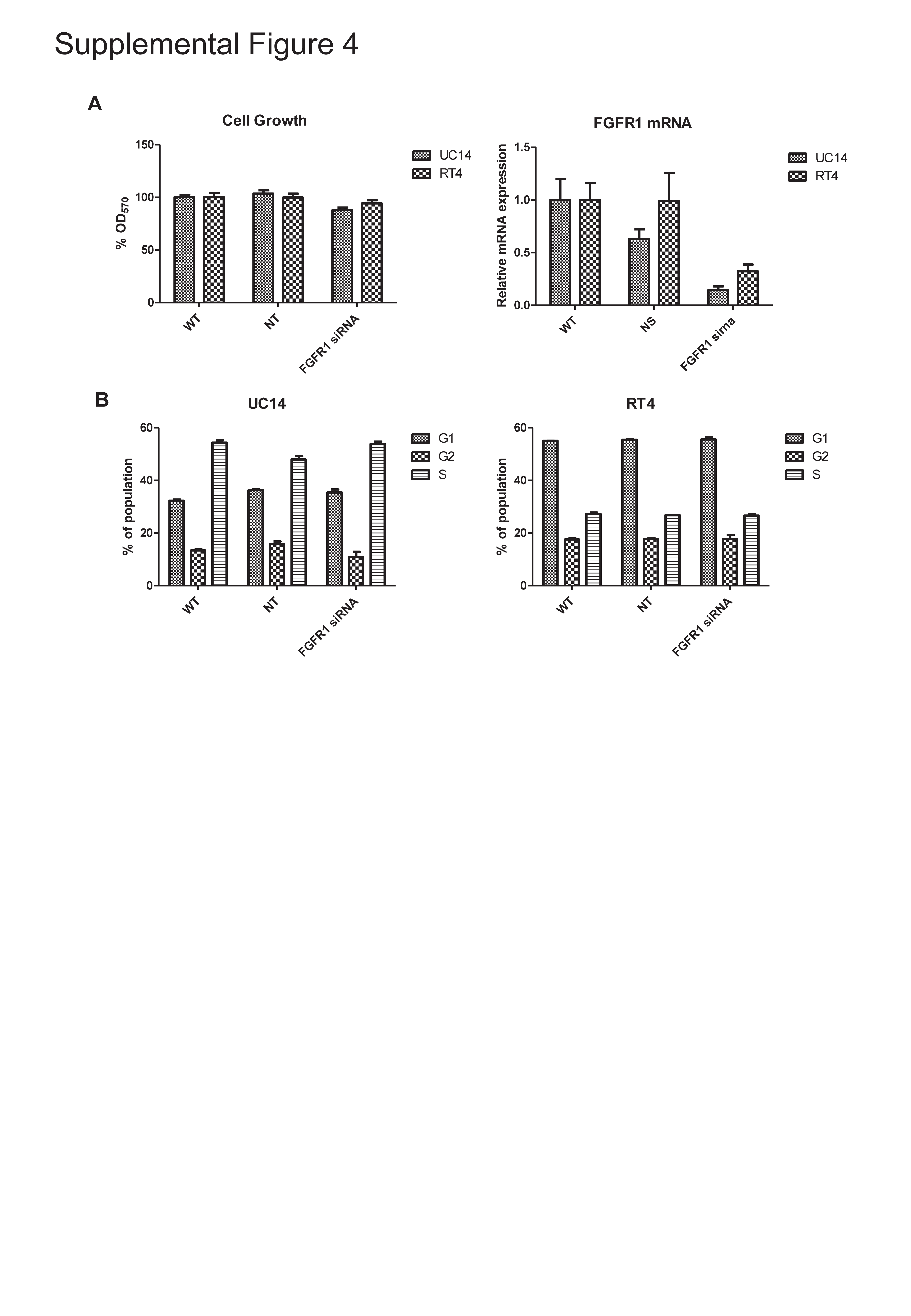

Supplement: Figure S4 — Effects of FGFR1 knockdown on FGFR1 expression and proliferation in RT4 and UM-UC14 cells. A. Left panel: UM-UC14 or RT4 cells were transiently transfected with either non-targeting (NT) or FGFR1-specific siRNAs and cell growth was measured at 48 h using MTT. Mean ± SEM, n = 8. Right Panel: the efficiency of FGFR1 silencing by siRNA was determined by quantitative RT-PCR. B. UM-UC14 or RT4 cells were transiently transfected with either non-targeting (NT) or FGFR1-specific siRNAs and percentages of cells within each phase of the cell cycle were quantified by propidium iodide staining and FACS analysis. Mean ± SEM, n = 3. (TIF) [file pone.0057284.s004.tif]

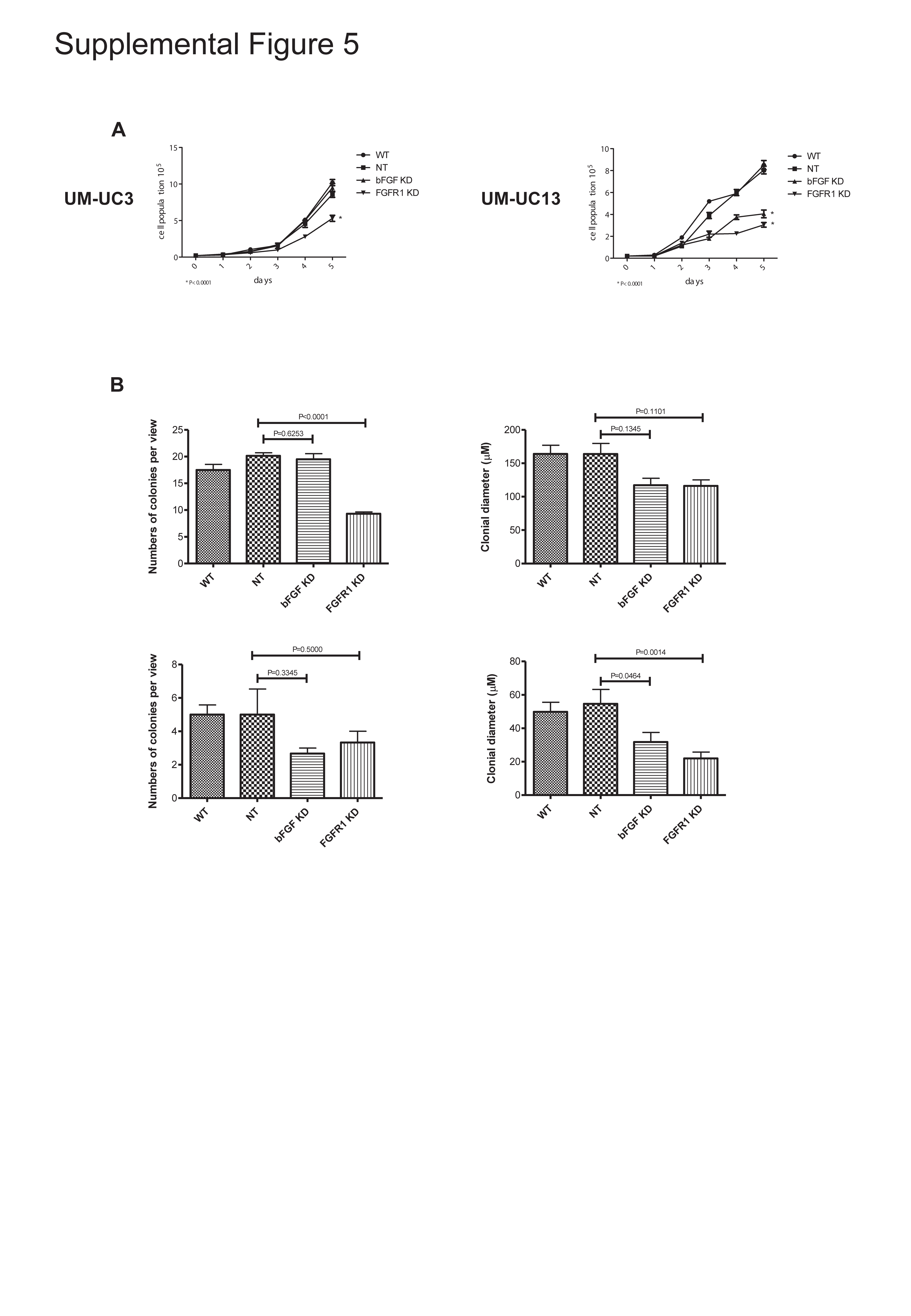

Supplement: Figure S5 — Effects of bFGF or FGFR1 knockdown in long-term assays. A. MTT results obtained in 5-day assays. Mean ± SEM, n = 6. *p<0.05. B. Results obtained in soft agar colony formation assays. The left panels display the numbers of colonies and the right panels colony diameters as determined by measuring colony growth in soft agar. Mean ± SEM, n = 5. (TIF) [file pone.0057284.s005.tif]

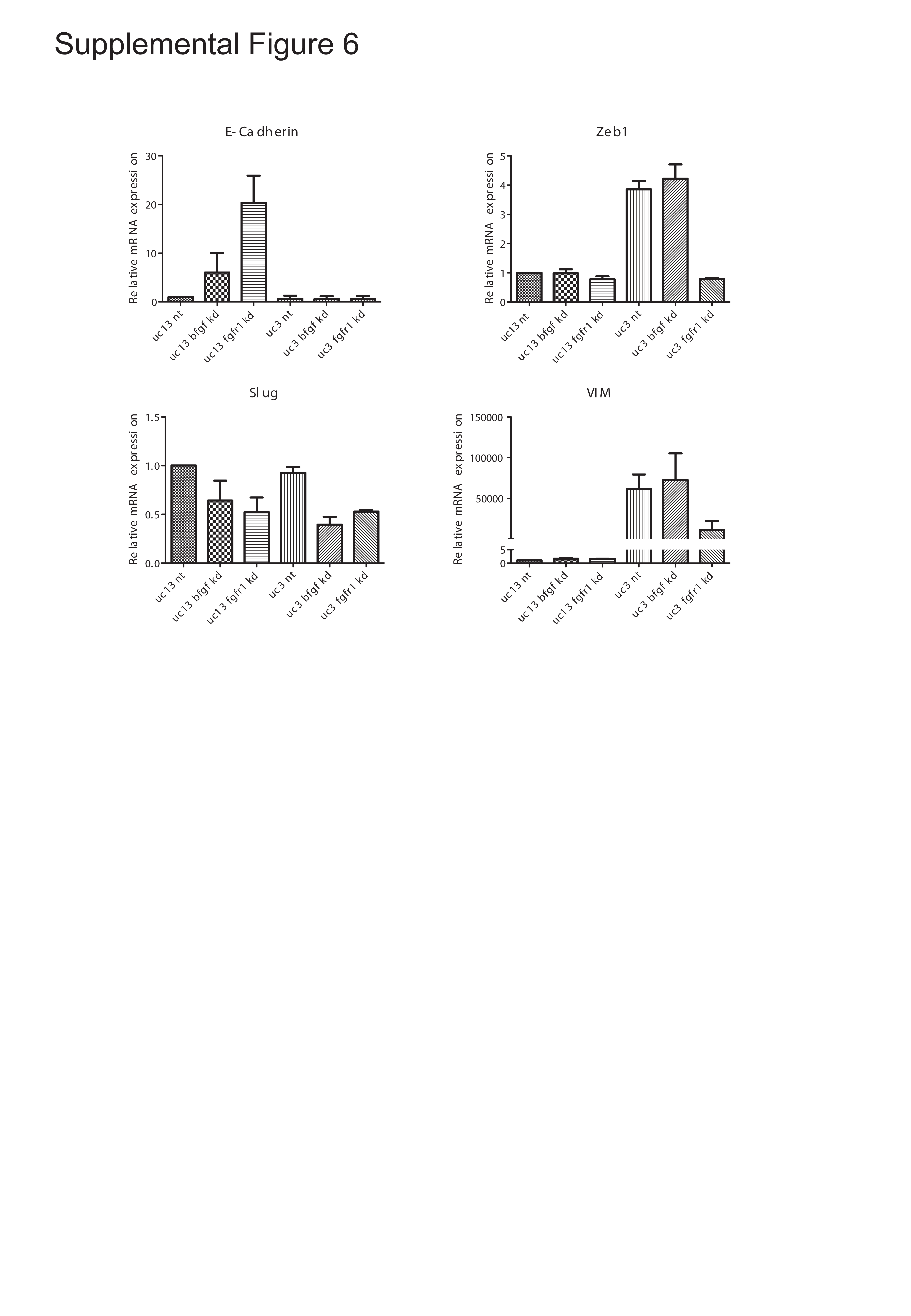

Supplement: Figure S6 — Effects of bFGF or FGFR1 knockdown on EMT marker expression. Mean ± SEM, n = 3. (TIF) [file pone.0057284.s006.tif]
